# A novel *MSMO1* variant presenting with severe psoriasiform dermatitis: A case from Saudi Arabia

**DOI:** 10.1016/j.jdcr.2026.08.041

**Published:** 2026-09-02

**Authors:** Nancy Shehata, Turki Alqarni, Husna Irfan Thalib, Zuhier Awan

**Affiliations:** a Department of Dermatology, King Abdullah Medical Complex, Jeddah, Saudi Arabia; b Department of Family Medicine, Ministry of National Guard Health Affairs, King Abdulaziz Medical City, AlAhsa, Saudi Arabia; c Family Medicine Physician, Makkah Healthcare Cluster, Makkah, Saudi Arabia; d General Medicine Practice Program, Batterjee Medical College, Jeddah, Saudi Arabia; e Department of Clinical Biochemistry, Faculty of Medicine, King Abdulaziz University, Jeddah, Saudi Arabia

**Keywords:** distal cholesterol biosynthesis disorder, inborn error of metabolism, MSMO1 deficiency, psoriasiform dermatitis, sterol-C4-methyl oxidase

## Introduction

*MSMO1* encodes sterol-C4-methyl oxidase, a key enzyme in distal cholesterol biosynthesis. Its deficiency causes accumulation of methylated sterol intermediates and relative cholesterol deficiency, disrupting epidermal barrier function and cellular signaling. Also known as sterol-C4-methyl oxidase deficiency, this rare inborn error of metabolism has multisystem manifestations. To date, only 12 molecularly confirmed cases have been reported; our patient represents the thirteenth.^1^^,^^2^

## Case report

The proband was a 5-year-old boy born at term to healthy first-degree cousins after an uncomplicated pregnancy. Microcephaly was noted at birth. Soon after delivery, hyperpigmented erosive plaques developed on the neck with an erythematous eczematous plaque in the genital area (Fig 1, *A*), progressing to chronic psoriasiform plaques involving the periorificial and genital regions (Fig 1, *B*).

**Fig 1:**
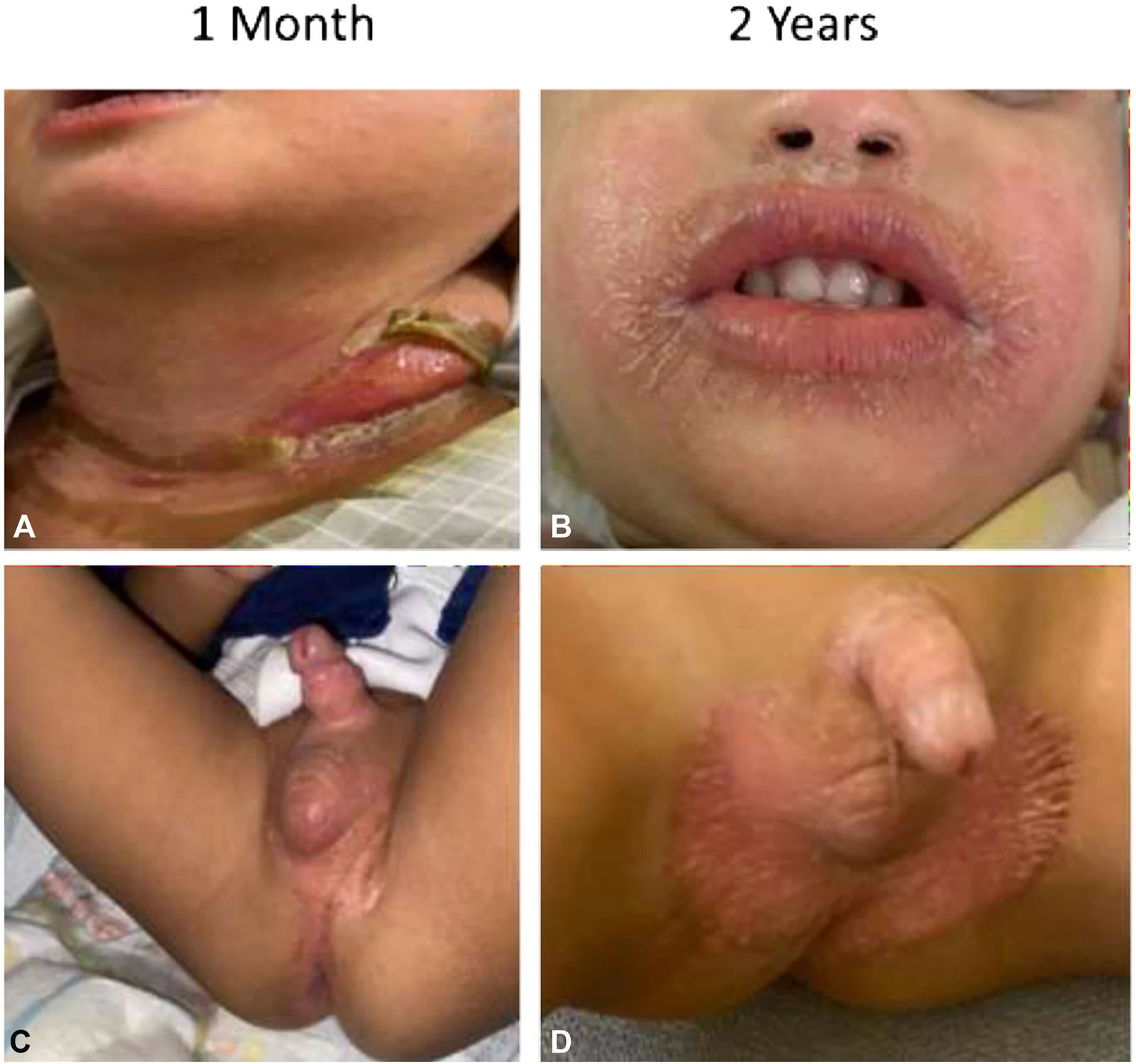
**A1,** At 1 month of age (*left*), hyperpigmented erosive plaques are noted over the cervical region, with a concomitant mildly erythematous, xerotic eczematous plaque involving the genital area. **B1,** By 2 years of age (*right*), there is evolution into well-demarcated, chronic psoriasiform plaques, predominantly distributed in periorificial, and genital regions.

Developmentally, he had delayed speech with articulation difficulties but appropriate social interaction. Neurological examinations were normal. Ophthalmologic evaluation showed ulcerative blepharitis without congenital cataract or other anterior segment abnormalities.

### Molecular findings

Whole-exome sequencing identified a novel homozygous methylsterol monooxygenase 1 (MSMO1) missense variant (NM_006,745.4.403T>C; p.Trp135Arg) in exon 3. The variant was absent from the 1000 Genomes Project and Genome Aggregation Database, and the affected tryptophan residue was highly conserved. Multiple in silico tools predicted a deleterious effect, including Sorting Intolerant From Tolerant, Polymorphism Phenotyping version 2, Combined Annotation Dependent Depletion, AlphaMissense, ClinPred, and Protein Variation Effect Analyzer. Segregation analysis demonstrated heterozygosity in both parents and 2 unaffected siblings, consistent with autosomal recessive inheritance (Fig 2), with no other phenotype-relevant variants identified. Based on PM2, PM3, PP3, and PP4, the variant met American College of Medical Genetics and Genomics/Association for Molecular Pathology 2015 criteria for *Likely Pathogenic*. A different pathogenic substitution at the same residue (p.Trp135Cys) further supports Trp135 as a critical functional site and application of PM5.

**Fig 2:**
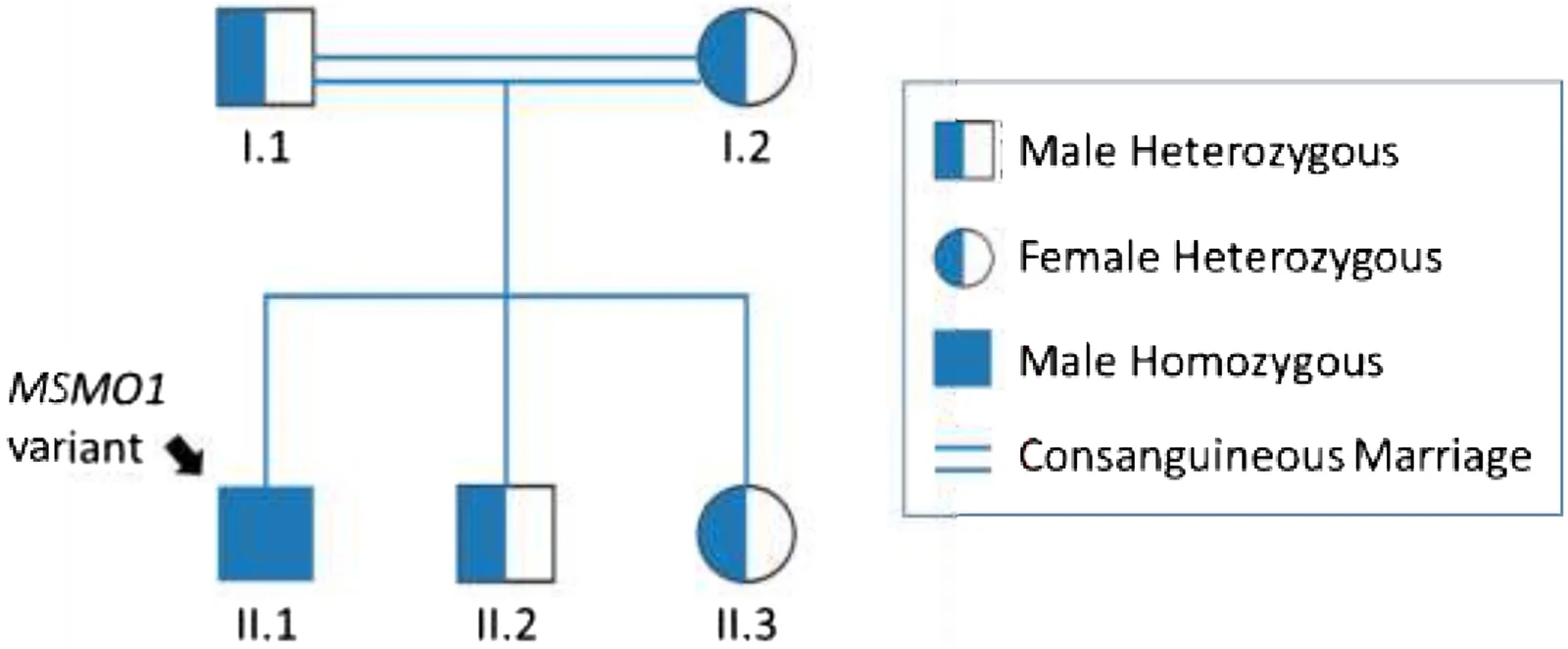
Pedigree illustrating familial segregation of the *MSMO1* variant. Both parents are heterozygous carriers, and segregation analysis demonstrates carrier status in 2 unaffected siblings, while 1 affected offspring is homozygous for the variant. This inheritance pattern is consistent with an autosomal recessive mode of transmission.

Skin biopsies performed on 2 occasions consistently showed psoriasiform acanthosis with elongated rete ridges, parakeratosis, preserved granular layer, and superficial chronic inflammation (Fig 3), supporting a chronic psoriasiform dermatosis and excluding alternative diagnoses. Normal serum zinc levels made zinc deficiency unlikely. Although serum alkaline phosphatase was not measured, the clinical phenotype, histopathology, normal zinc level, and confirmatory molecular findings were not consistent with acrodermatitis enteropathica or other nutritional deficiency disorders. The lack of alkaline phosphatase measurement is acknowledged as a limitation.

**Fig 3:**
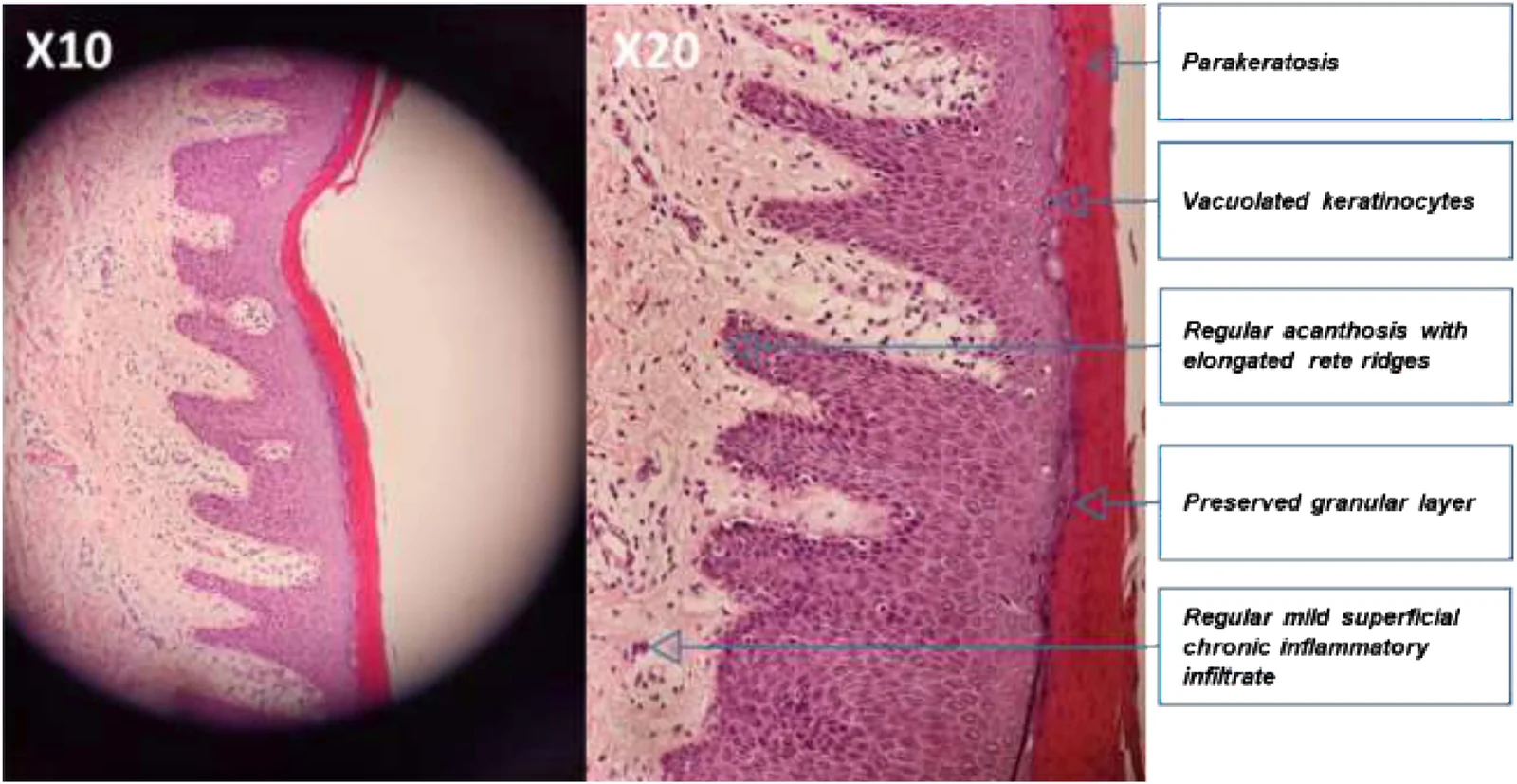
Histopathologic examination of lesional skin demonstrating regular psoriasiform acanthosis with elongated rete ridges, compact orthokeratosis, preservation of the granular layer, scattered vacuolated keratinocytes showing koilocytic-like change, and a mild superficial chronic inflammatory infiltrate. These findings are consistent with a chronic psoriasiform inflammatory dermatosis.

### Management and outcome

The diagnosis and therapeutic rationale were discussed in detail with the parents, and targeted metabolic therapy was initiated based on previously reported therapeutic approaches in MSMO1 deficiency.^1^, ^2^, ^3^, ^4^, ^5^, ^6^, ^7^, ^8^ Baseline biochemical evaluation demonstrated normal serum cholesterol fractions and bile acid levels. (Table I).

**Table I:** Baseline lipid profile and bile acid levels prior to initiation of therapy

Laboratory test	Result	Unit	Reference range	Interpretation
Total bile acids (serum)	2.7	μmol/L	0-10	Within normal range
Cholesterol (total)	3.48	mmol/L	<4.4	Within normal range
HDL cholesterol	1.63	mmol/L	>1.45	Within normal range
LDL cholesterol	1.72	mmol/L	<2.59	Within normal range
7- dehydrocholesterol	1.01	μmol/L	<2.86	Within normal range

*HDL*, High-density lipoprotein; *LDL*, low-density lipoprotein.

Based on the molecular diagnosis and characteristic dermatologic phenotype, the patient received oral atorvastatin (10 mg daily), topical atorvastatin,^3^ and dietary cholesterol supplementation. Before treatment, extensive erythematous, scaly, fissured plaques predominantly involved the flexural and periorificial regions (Fig 4, *A*). After 8 weeks, erythema, scaling, and plaque thickness markedly improved with partial re-epithelialization (Fig 4, *B*). Sustained improvement was observed at 3 months; however, temporary treatment interruption led to partial relapse, consistent with persistent metabolic dysfunction (Fig 4, *C*).

**Fig 4:**
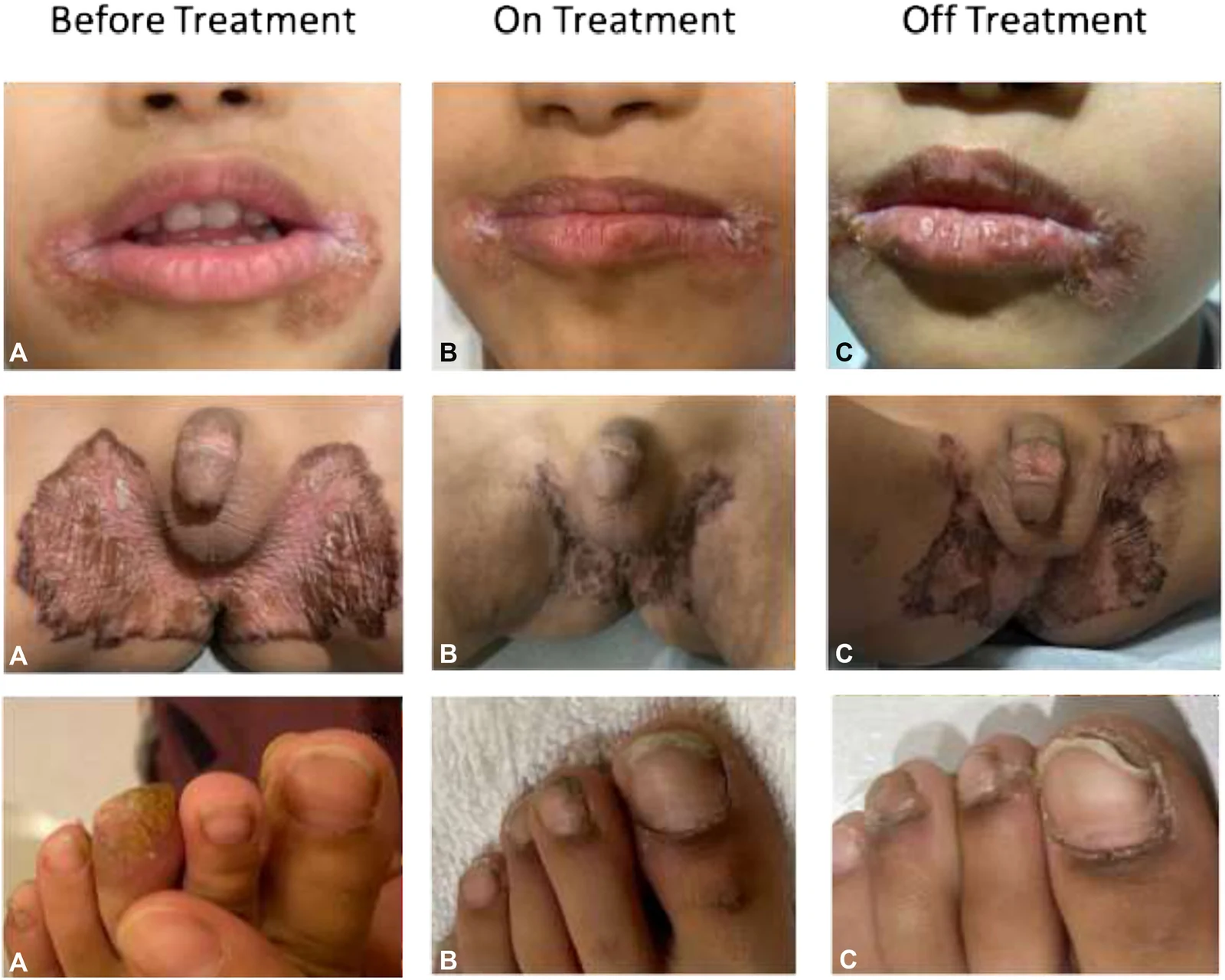
Comparison of clinical images at 5 years of age a before treatment, after 8 weeks on treatment, and 3 months off treatment. **A,** Shows well-developed, chronic psoriasiform plaques mainly affecting the periorificial, flexural, and genital areas, with noticeable periungual thickening. **B,** After 8 weeks on treatment, there was clear improvement, with reduced redness and scaling, flatter plaques, and some remaining post-inflammatory hyperpigmentation. **C,** Three months off treatment, a partial relapse was observed, characterized by recurrent erythema, scaling, and superficial erosions, consistent with renewed disease activity following treatment interruption.

## Discussion

MSMO1 deficiency is a rare distal cholesterol biosynthesis disorder caused by impaired sterol-C4-methyl oxidase activity, resulting in accumulation of methylated sterol intermediates and disrupted epidermal lipid homeostasis, which drives psoriasiform dermatitis.^3^, ^4^, ^5^ Reported cases show considerable phenotypic heterogeneity, particularly in neurodevelopmental and ocular involvement, likely reflecting residual enzyme activity, variant location, and modifying factors.^3^, ^4^, ^5^

Compared with previously reported patients who often had congenital cataracts and marked neurological impairment, our patient presented predominantly with severe early-onset dermatitis, mild developmental delay, and normal ocular examination, supporting a broader phenotypic spectrum with possible cutaneous predominance in some genotypes.^6^^,^^7^ Skin biopsies consistently showed psoriasiform acanthosis with elongated rete ridges, parakeratosis, preserved granular layer, and superficial chronic inflammation, correlating with the clinical phenotype and providing additional support for MSMO1-associated psoriasiform dermatitis.

The recently reported p.Trp135Cys variant affects the same highly conserved Trp135 residue as the p.Trp135Arg variant identified in our patient. Two independent pathogenic missense substitutions at the same codon support the functional importance of Trp135 and provide additional evidence for pathogenicity.

Segregation analysis demonstrated homozygosity in the proband and heterozygosity in both parents and siblings, consistent with autosomal recessive inheritance. Rapid improvement with statin therapy and relapse after interruption supported the diagnosis and emphasized the need for sustained treatment.^3^^,^^4^ Although specialized profiling of diagnostic 4α-methyl- and 4,4-dimethylsterol intermediates was unavailable despite normal serum 7-dehydrocholesterol (Table I), the phenotype, molecular findings, segregation analysis, and therapeutic response strongly supported MSMO1 deficiency. Since the first molecularly confirmed cohort by He et al in 2014, the phenotypic spectrum has expanded to include variable dermatologic, ocular, and neurodevelopmental manifestations (Table II).^1^, ^2^, ^3^, ^4^, ^5^, ^6^, ^7^

**Table II:** Summary of molecularly characterized individuals with MSMO1 deficiency reported in the literature

Patient	Author	Year	Age at presentation	Consanguinity	Major dermatologic phenotype	Neurological severity	Ocular findings	*MSMO1* variant∗	Metabolic therapy
1	He et al^1^	2014	Childhood	Yes	Inflammatory or psoriasiform cutaneous manifestations with variable severity	Developmental delay	Congenital cataract	Biallelic *MSMO1* pathogenic variants (compound heterozygous or homozygous, as originally reported)	Statin + dietary cholesterol + bile acids
2			Childhood	Yes					Statin + dietary cholesterol
3			Infancy	Yes					Statin + dietary cholesterol
4			Childhood	Yes					Statin + dietary cholesterol
5	Frisso et al^2^	2017	19 y	No	Mild sebopsoriasiform dermatitis	Developmental delay	Congenital cataract	Compound heterozygous p.Tyr244Cys; p.Gly202Glu	-
6	Kalay Yildizhan et al^3^	2020	Childhood	Yes	Severe psoriasiform dermatitis	Severe developmental delay	Optic hypoplasia/nystagmus	Homozygous p.Asn27Thr	Statin + dietary cholesterol + topical cholesterol/statin
7	Kalay Yildizhan et al^3^	2020	Childhood	Yes	Severe psoriasiform dermatitis	Severe developmental delay	Optic nerve hypoplasia	Homozygous p.Asn27Thr	Statin + dietary cholesterol + topical cholesterol/statin
8	Morales et al^4^	2022	Infancy	No	Intermittent perianal dermatitis progressing to generalized plaques	Developmental delay	Congenital cataract	Maternally inherited VUS, (p. Pro179Leu), and a paternally inherited likely pathogenic variant, (p.Tyr244Cys)	Statin
9	Tkemaladze et al^5^	2023	Childhood	Yes	Psoriasiform dermatitis with alopecia universalis	Severe developmental delay	None	Homozygous p.Glu183Ala	Statin + dietary cholesterol + bile acids
10	Tkemaladze et al^5^	2023	Childhood	Yes	Psoriasiform dermatitis with alopecia universalis	Severe developmental delay		Homozygous p.Glu183Ala	Statin + dietary cholesterol + bile acid + topical cholesterol/statin
11	Null et al^6^	2025	Pediatric	Not stated	Inflammatory dermatitis phenotype	Developmental delay	Cataract	Compound heterozygous *MSMO1* variants: (p.Trp135Cys); (p.Trp267Ter)	Statin + dietary cholesterol
12	Hassas et al^7^	2026	Infancy	Not stated	Segmental confluent hyperpigmentation of his right trunk	Developmental delay	Infantile cataract	Homozygous missense	Dietary cholesterol
13	Present case	2026	5 y	Yes	Cutaneous erosion that evolved to chronic psoriasiform dermatitis	Developmental delay, microcephaly	Blepharitis	Homozygous p.Trp135Arg	Statin + dietary cholesterol + topical statin

*VUS*, Variant of uncertain significance.

∗ Variant nomenclature is reported according to the original publications. Human Genome Variation Society complementary DNA annotations were included where available; otherwise, protein-level nomenclature was retained to avoid introducing unsupported annotations.

Despite extensive first-pass metabolism, the lipophilic nature of atorvastatin permits distribution to peripheral tissues, including the skin. In MSMO1 deficiency, oral atorvastatin suppresses cholesterol synthesis and toxic 4-methylsterol intermediates, while cholesterol supplementation restores the deficient end product through physiological feedback inhibition. Topical atorvastatin further reduces epidermal sterol accumulation and improves barrier function with minimal systemic exposure, paralleling topical lovastatin–cholesterol therapy in congenital hemidysplasia with ichthyosiform nevus and limb defects syndrome. The favorable response in our patient supports this combined pathway-directed approach.^1^, ^2^, ^3^^,^^7^^,^^8^

To date, 12 molecularly confirmed cases of MSMO1 deficiency have been reported; our patient represents the thirteenth. Most present in infancy or childhood, often in consanguineous families, with psoriasiform dermatitis, variable neurodevelopmental impairment, and frequent but inconsistent congenital cataracts. Limitations include the lack of biochemical sterol profiling and limited follow-up. However, the phenotype, segregation pattern, and therapeutic response strongly support the diagnosis and expand the predominantly cutaneous spectrum of MSMO1 deficiency.

## Conclusion

This is the first reported Saudi Arabian case of MSMO1 deficiency, caused by a novel homozygous variant with a predominantly cutaneous phenotype. It demonstrates that the disorder may present primarily with dermatologic manifestations despite minimal neurological or ocular involvement. The marked response to oral atorvastatin, topical atorvastatin, and dietary cholesterol supplementation highlights the value of early diagnosis and targeted metabolic therapy in improving cutaneous disease and potentially altering disease course.

## Conflicts of interest

None disclosed.

## References

[1] He M., Kratz L.E., Michel J.J. (2011). Mutations in the human SC4MOL gene encoding a methyl sterol oxidase cause psoriasiform dermatitis, microcephaly, and developmental delay. J Clin Invest.

[2] Frisso G., Gelzo M., Procopio E. (2017). A rare case of sterol-C4-methyl oxidase deficiency in a young Italian male: biochemical and molecular characterization. Mol Genet Metab.

[3] Kalay Y.I., Gökpınar Ili E., Onoufriadis A. (2020). New homozygous missense MSMO1 mutation in two siblings with SC4MOL deficiency presenting with psoriasiform dermatitis. Cytogenet Genome Res.

[4] Morales J.A., Curry C.J., Tise C.G., Kratz L., Enns G.M. (2022). Clinical characterization of a new individual with mild SC4MOL deficiency: diagnostic and therapeutic implications. J Transl Genet Genom.

[5] Tkemaladze T., Bratland E., Bregvadze K. (2023). MSMO1 deficiency: a potentially partially treatable ultrarare neurodevelopmental disorder with psoriasiform dermatitis, alopecia and polydactyly. Clin Dysmorphol.

[6] Null E., Beckman E., Lam C. (2025). SC4MOL deficiency: 11th reported patient with updates in treatment outcomes and review of reported cases. GIM Open.

[7] Hassas N., Drackley A., Ivanisevic J., Ranaivo H.R., Kurup S.P. (2026). Infantile cataracts associated with a homozygous missense MSMO1 variant: case report and literature review. Reports.

[8] Paller A.S., van Steensel M.A., Rodriguez-Martín M. (2011). Pathogenesis-based therapy reverses cutaneous abnormalities in an inherited disorder of distal cholesterol metabolism. J Invest Dermatol.

